# Supplementary material for: Plants Specifically Modulate the Microbiome of Root-Lesion Nematodes in the Rhizosphere, Affecting Their Fitness
Source: Microorganisms. 2021 Mar 25;9(4):679. doi: 10.3390/microorganisms9040679 (PMC8064444; doi:10.3390/microorganisms9040679)
Supplement: Supplementary file 1 [file microorganisms-09-00679-s001.pdf]

microorganisms

MDPI

Supplement

Plants specifically modulate the microbiome of root-lesion nematodes in the rhizosphere, affecting their fitness

Ahmed Elhady [1,2,*], Olivera Topalović [1] and Holger Heuer [1]

[1] Institute for Epidemiology and Pathogen Diagnostics, Julius Kühn Institute (JKI) – Federal Research Centre for Cultivated Plants, Braunschweig, Germany; otopalovic@agro.au.dk (O.T); holger.heuer@julius-kuehn.de (H.H)
[2] Department of Plant Protection, Faculty of Agriculture, Benha University, Benha, Egypt
* Correspondence: ahmed.gomaa@julius-kuehn.de/ahmed.elhady1985@gmail.com

Table S1. Percentange dissimilarity of bacterial or fungal communities attached to the cuticle of *Pratylenchus penetrans* that were incubated in soil suspensions from different rhizospheres or bulk soil, or non-attached microbial communities in the respective soil suspension.

	Sources of Soil Suspensions of the Pairwise Comparisons of Bacterial or Fungal DGGE Fingerprints	Dissimilarity (%) [a]			
		Bacteria		Fungi	
		Soil	Attached to Cuticle	Soil	Attached to Cuticle
Experiment (1)	Bulk soil vs. maize rhizosphere	37	23	58	62
	Bulk soil vs. tomato rhizosphere	64	35	35	88
	Bulk soil vs. soybean rhizosphere	30	40	49	65
	Maize vs. tomato rhizosphere	65	43	31	87
	Maize vs. soybean rhizosphere	14	51	61	43
	Soybean vs. tomato rhizosphere	51	68	30	57
Experiment (2)	Bulk soil vs. maize rhizosphere	32	34	33	14
	Bulk soil vs. oat rhizosphere	29	31	44	28
	Bulk soil vs. Ethiopian mustard rhizosphere	31	12	18	10
	Maize vs. oat rhizosphere	12	29	28	29
	Maize vs. Ethiopian mustard rhizosphere	26	36	27	17
	Oat vs. Ethiopian mustard rhizosphere	25	22	35	3

[a] d-value: average of pairwise Pearson correlation coefficients among DGGE fingerprints within each group minus average of pairwise Pearson correlation coefficients among DGGE fingerprints of different groups.

Table S2. Identification and frequency of fungal and bacterial species associated with *Pratylenchus penetrans* after baiting in suspensions of bulk soil or different rhizosphere soils.

	Band	Closest Genbank match	GenBank accession no. (% identity)	Specificity of band				
				Bulk soil	Maize rhizo-sphere	Soybean rhizo-sphere	Tomato rhizo-sphere	In-oculum
Fungal DGGE	1	*Malassezia restricta*	CP030254.1 (99%)		X	X	X	
	2	*Penicillium corylophilum*	MF475910.1 (99%)		X	X		
	3	*Penicillium digitatum*	MH864871.1 (99%)	X				
	4	*Acremonium psychrophilum*	MH862386.1 (96%)	X				
	5	*Simplicillium sympodiophorum*	KY434158.1 (99%)	X				
	6	*Malassezia globosa*	KM269155.1 (99%)	X	X			
	7	*Myrothecium verrucaria*	FJ235085.1 (99%)		X			
	8	*Penicillium allii*<br>*Penicillium gladioli*<br>*Penicillium hordei*	AF218787.1 (99%)<br>MH856256.1 (99%)<br>MH859204.1 (100%)				X	
	9	*Scoliciosporum umbrinum*	KX133008.1 (99%)	X				
	10	*Aspergillus tonophilus*	MH858639.1 (100%)		X			
	11	*Cladosporium tenuissimum*	MG569541.1 (99%)	X	X	X	X	X
	12	*Cladosporium cladosporioides*<br>*Cladosporium allicinum*	MH790419.1(100%)<br>KY420929.1 (99%)			X	X	
	13	*Sporidiobolus pararoseus*	KY105483.1 (100%)				X	
	14	*Cutaneotrichosporon curvatus*	KY102995.1 (100%)				X	
Bacterial DGGE	1	*Paraburkholderia dipogonis*	NR_145902.1 (100%)			X		
	2	*Cutibacterium acnes*	NR_040847.1 (99%)	X	X	X	X	X
	3	*Pseudomonas guariconensis*	NR_135703.1 (99%)		X		X	
	4	*Pseudomonas putida*	NR_114794.1 (99%)			X	X	
	5	*Pantoea stewartii*	NR_104928.1 (95%)		X	X		
	6	*Bradyrhizobium embrapense*	NR_145861.1 (95%)	X	X	X	X	
	7	*Streptococcus thermophilus*	NR_042778.1 (99%)	X				
	8	*Pseudomonas synxantha*	NR_113583.1 (100%)	X	X	X	X	
	9	*Streptococcus rubneri*	NR_109720.1 (99%)				X	
	10	*Enterobacter xiangfangensis*	NR_126208.1 (99%)	X			X	
	11	*Granulicatella adiacens Acinetobacter lwoffii*	NR_025862.1 (99%)<br>NR_113346.1 (99%)	X			X	
	12	*Streptococcus himalayensis*	NR_156072.1 (99%)	X	X	X	X	
	13	*Moraxella nonliquefaciens*	NR_104938.1 (99%)				X	
	14	*Haemophilus sputorum*<br>*Veillonella tobetsuensis*	NR_118143.1 (99%)<br>NR_113570.1 (93%)	X	X	X		
	15	*Streptococcus mitis*<br>*Streptococcus salivarius*	NR_116207.1 (99%)<br>NR_042776.1 (100%)	X	X	X		

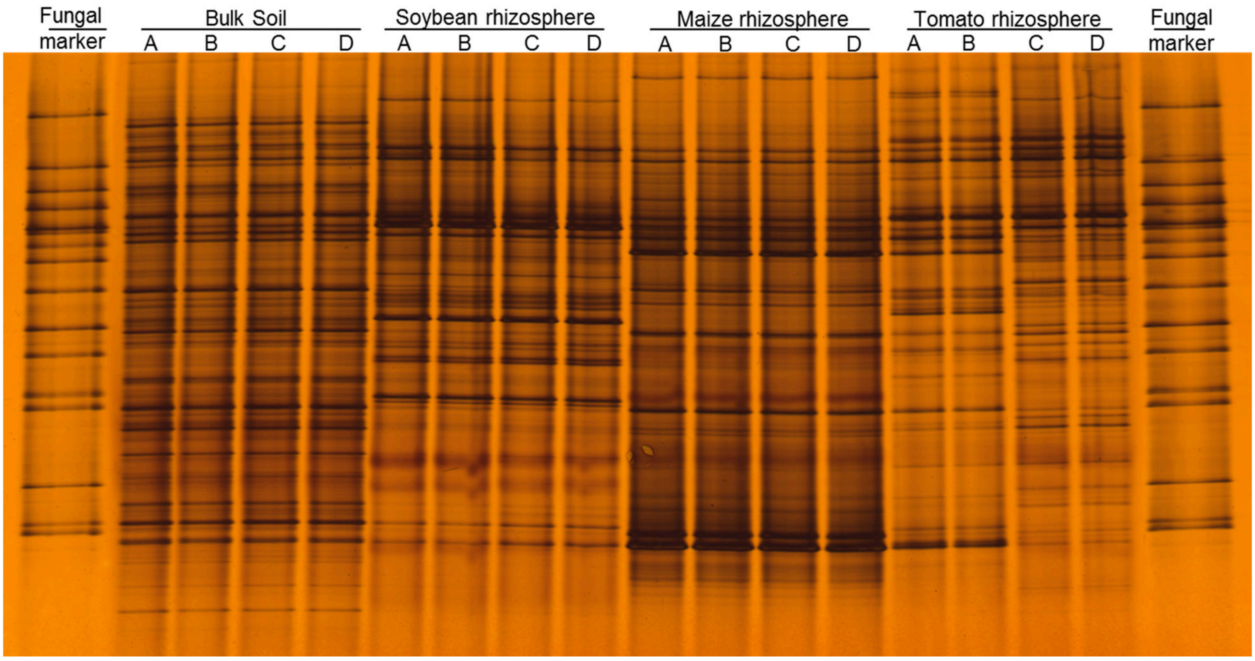

Figure S1. DGGE profiles of fungal ITS fragments amplified from DNA of bulk soil and rhizospheres of maize, soybean, and tomato plants grown in the same soil. Letters A, B, C, and D represent biological replicates of each treatment.

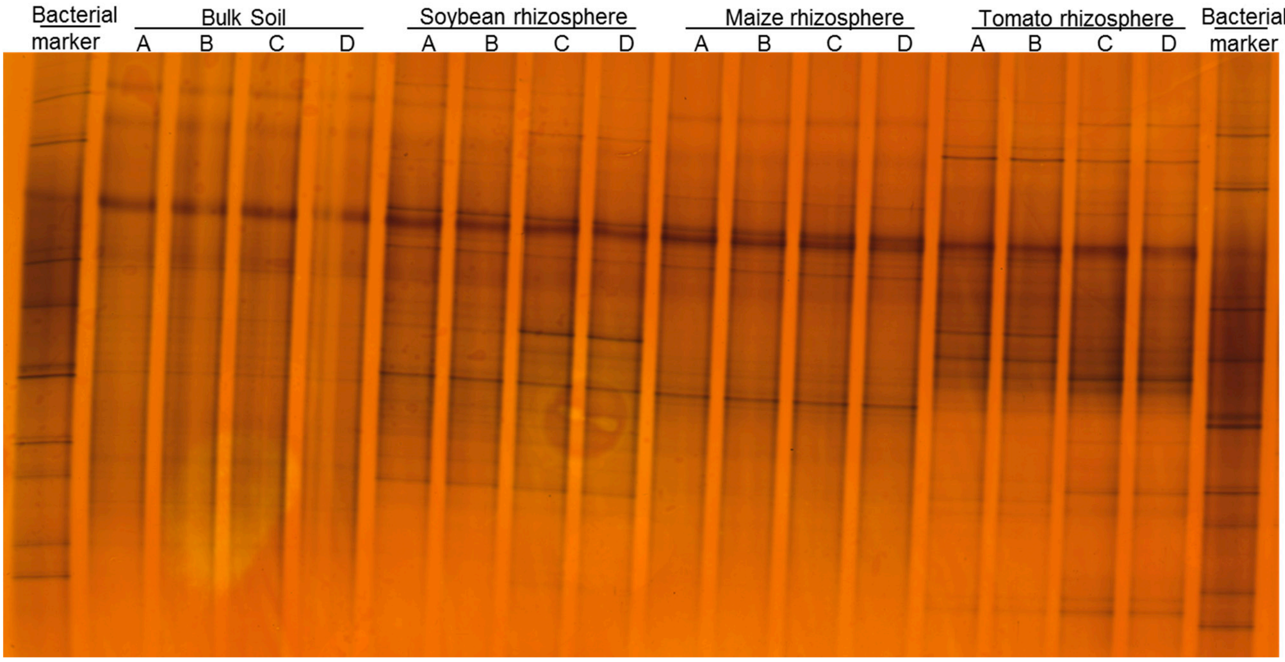

Figure S2. DGGE profiles of bacterial 16S rRNA fragment fragments amplified from DNA of bulk soil and rhizoScheme 3. Box-PCR fingerprint of bacterial strains isolated from *Pratylenchus penetrans* cuticle after incubation in bulk soil or the rhizosphere soils of maize, tomato, or soybean.

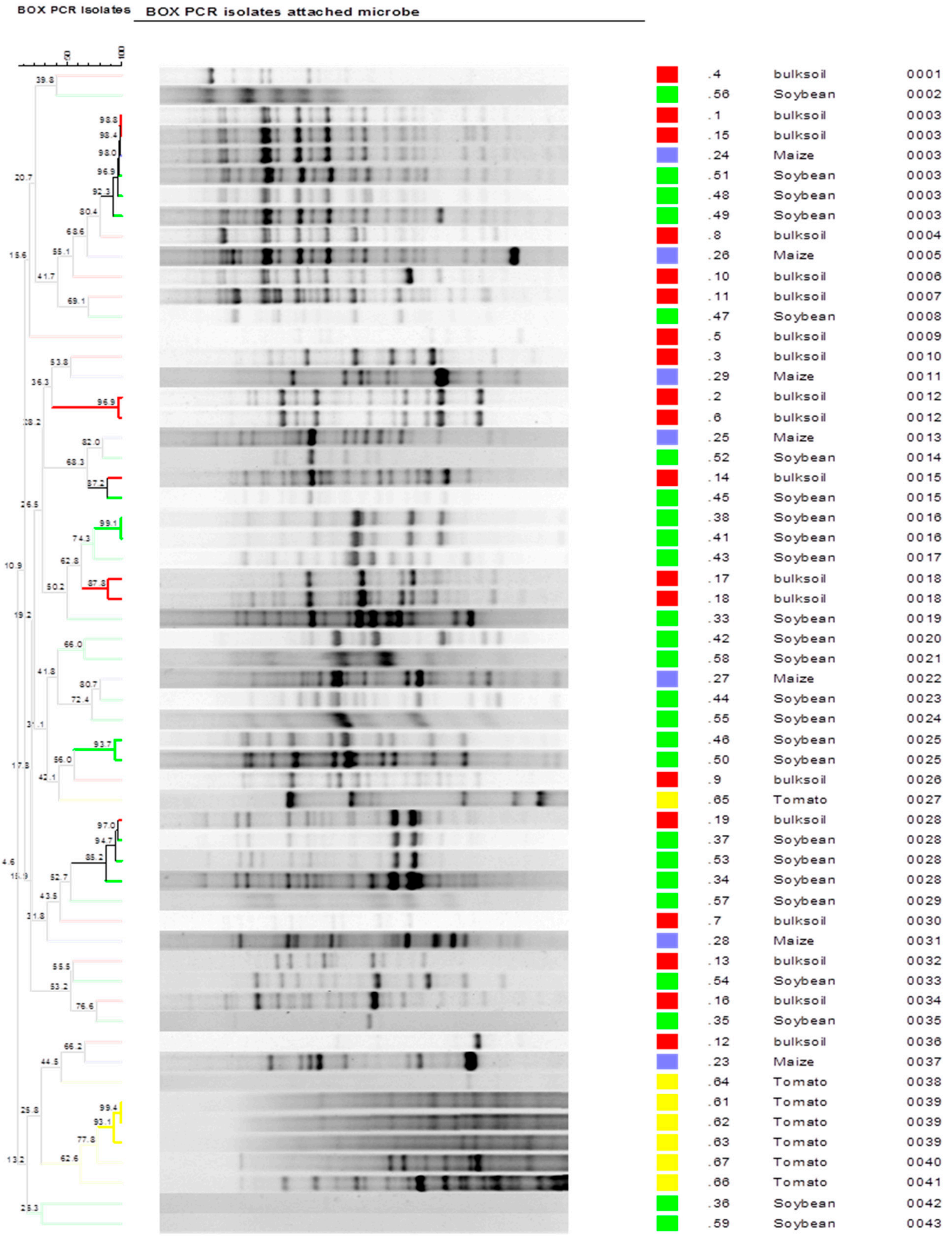

Figure S3. Box-PCR fingerprint of bacterial strains isolated from *Pratylenchus penetrans* cuticle after incubation in bulk Scheme 4. Effect of pre-incubation of *Pratylenchus penetrans* in root exudates of soybean, maize, or tomato on the bacterial community attached to the cuticle. Contr.: control DNA from surface disinfected nematodes that served as inoculum.

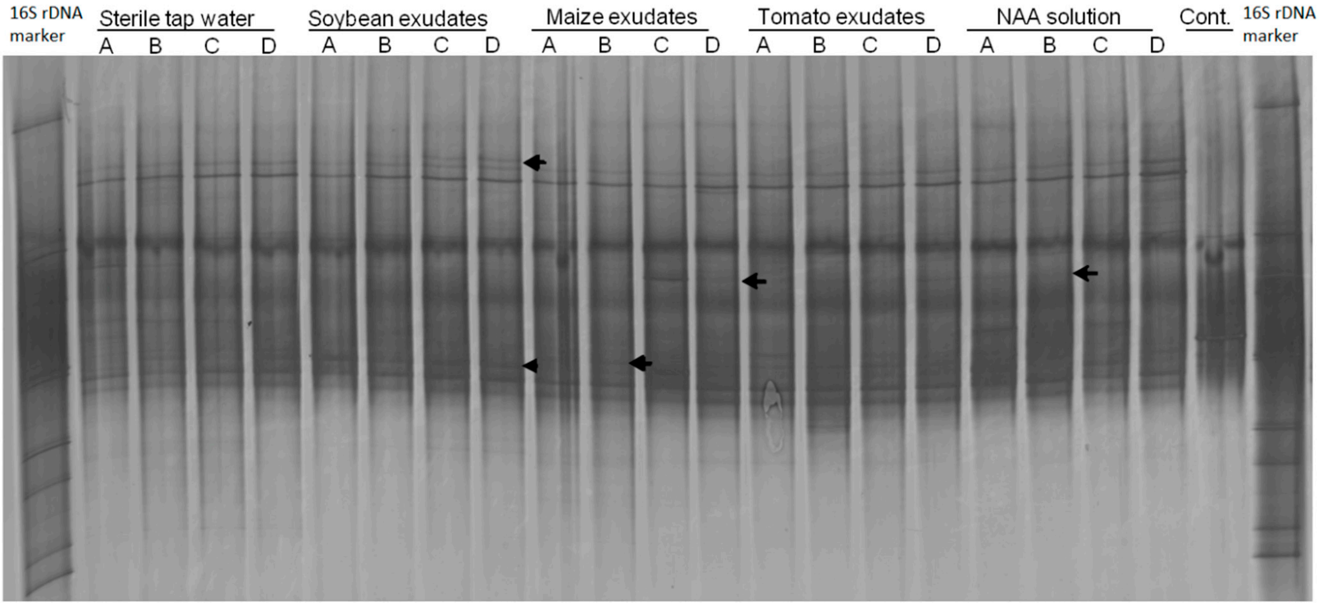

Figure S4. Effect of pre-incubation of *Pratylenchus penetrans* in root exudates of soybean, maize, or tomato on the bacterial community attached to the cuticle. Contr.: control DNA from surface disinfected nematodes that served as inoculum.

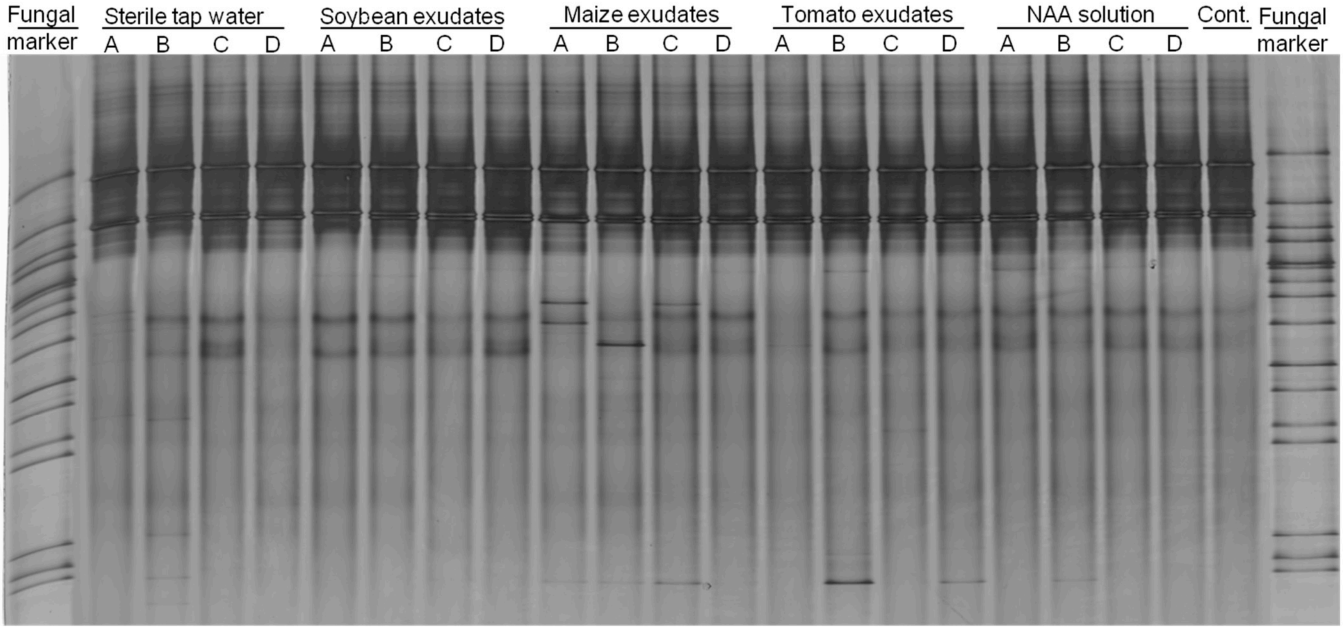

Figure S5. Effect of pre-incubation of *Pratylenchus penetrans* in root exudates of soybean, maize, or tomato on the fungal community attached to the cuticle. NAA: 1 μM α-naphthalene acetic acid (auxin). Contr.: control DNA from surface disinfected nematodes that served as inoculum.
